# A preclinical microbeam facility with a conventional x‐ray tube

**DOI:** 10.1118/1.4966032

**Published:** 2016-11-02

**Authors:** Stefan Bartzsch, Craig Cummings, Stephan Eismann, Uwe Oelfke

**Affiliations:** ^1^ Institute of Cancer Research, 15 Cotswold Road, Belmont Sutton, Surrey SM2 5NG, United Kingdom; ^2^ Department of Physics and Astronomy, University of Heidelberg, Grabengasse 1, 69117 Heidelberg, Germany; ^3^ Institute of Cancer Research, 15 Cotswold Road, Belmont Sutton, Surrey SM2 5NG, United Kingdom

**Keywords:** biomolecular effects of radiation, cancer, cellular effects of radiation, DNA, dosimetry, radiation therapy, tumours, X‐ray applications, Therapeutic applications, including brachytherapy, DNA, Cancer, Cell processes, Effects of ionizing radiation on biological systems, Dosimetry/exposure assessment, Radiation therapy, Scintigraphy, microbeam radiation therapy, compact microbeam sources, dosimetry, collimators, cellular response, Collimators, Dosimetry, Vacuum tubes, Photons, Cancer, Intracellular signaling, Magnetic resonance imaging, Tissues, Synchrotron radiation, Radiotherapy sources

## Abstract

**Purpose:**

Microbeam radiation therapy is an innovative treatment approach in radiation therapy that uses arrays of a few tens of micrometer wide and a few hundreds of micrometer spaced planar x‐ray beams as treatment fields. In preclinical studies these fields efficiently eradicated tumors while normal tissue could effectively be spared. However, development and clinical application of microbeam radiation therapy is impeded by a lack of suitable small scale sources. Until now, only large synchrotrons provide appropriate beam properties for the production of microbeams.

**Methods:**

In this work, a conventional x‐ray tube with a small focal spot and a specially designed collimator are used to produce microbeams for preclinical research. The applicability of the developed source is demonstrated in a pilot *in vitro* experiment. The properties of the produced radiation field are characterized by radiochromic film dosimetry.

**Results:**

50 *μ*m wide and 400 *μ*m spaced microbeams were produced in a 20 × 20 mm^2^ sized microbeam field. The peak to valley dose ratio ranged from 15.5 to 30, which is comparable to values obtained at synchrotrons. A dose rate of up to 300 mGy/s was achieved in the microbeam peaks. Analysis of DNA double strand repair and cell cycle distribution after *in vitro* exposures of pancreatic cancer cells (Panc1) at the x‐ray tube and the European Synchrotron leads to similar results. In particular, a reduced G2 cell cycle arrest is observed in cells in the microbeam peak region.

**Conclusions:**

At its current stage, the source is restricted to *in vitro* applications. However, moderate modifications of the setup may soon allow *in vivo* research in mice and rats.

## 1. INTRODUCTION

In radiotherapy, as in any other cancer treatment, the aim is to maximize its lethal effect to the tumor tissue while reducing side effects to surrounding healthy tissue as much as possible. Conventionally this aim is achieved by geometrically concentrating lethal dose levels in the tumor and exposing organs at risk below the tissue tolerance.

In microbeam radiation therapy (MRT), the tumor is irradiated by arrays of micrometer wide planar beams of unconventionally high doses of up to a few hundred Grays that are separated by several hundred micrometer wide low dose regions.^1^ These treatment fields selectively destroy tumor tissue^2^, ^3^, ^4^ while leaving penetrated healthy tissue unimpaired.^5^, ^6^ The remarkable resistance of normal tissue to microbeams has been demonstrated in various preclinical studies, beginning with neutron beams in the 1960s (Refs. 7, 8, 9, 10) to synchrotron generated photon beams in the last 25 years.^2^, ^5^, ^6^, ^11^, ^12^

Various biological mechanisms have been suggested to facilitate this microbeam effect. Manifold evidence indicates that blood vessels and their different repair efficiencies in malignant and healthy tissue are essential to explain the differential effect of microbeams.^2^, ^4^, ^5^, ^13^ Apart from that experiments demonstrate that bystander signals,^14^, ^15^ changes in the immune response, DNA repair, and variations in the cell cycle^16^ are important for the biological response to MRT.

Currently, the production of microbeams can only be facilitated at large synchrotron facilities as the European Synchrotron (ESRF) in Grenoble (France). This strongly limits preclinical research with microbeams and therefore delays their clinical application. MRT requires almost parallel beams at photon energies of around 100 keV to ensure sharp beam penumbras and extremely high dose rates to avoid degradation of the beam profiles due to respiratory or cardiovascular motion.^17^ There are several attempts to move toward alternative microbeam sources, such as laser accelerated protons^18^ and inverse Compton scattering.^19^ Also x‐ray tubes have been suggested as a microbeam source.^20^ Hadsell *et al.* ^21^ experimentally realized a preclinical setup with carbon nanotube x‐ray tubes and produced 300 *μ*m wide microbeams with a dose rate of 21.7 mGy/s per nanotube cathode. At a source to target distance of apparently 25.4 cm, a PVDR of 17 was measured for four 1.4 mm spaced beams at 1.27 mm depth in PMMA. Although this is a major step forward to compact microbeam sources, dose rates are still significantly smaller and beam widths larger than those achieved with a synchrotron.

In this study, we explore the possibility to use a conventional x‐ray tube to produce microbeams for preclinical studies. The applicability of the developed source for preclinical research in MRT is demonstrated in an *in vitro* experiment and compared to experiments at the ESRF. The properties of the produced MRT field are analyzed and discussed.

## 2. METHODS

### 2.A. Setup of the microbeam source

Naturally, synchrotrons are better suited for the production of microbeams than x‐ray tubes. The brilliance of synchrotrons defined as emitted photons per area, time, and solid angle is ten orders of magnitude higher than for conventional x‐ray tubes. Therefore, the synchrotron produces highly nondivergent almost perfectly parallel beams. These beams can easily be collimated by an array of parallel slits resulting in microbeams with sharp penumbras whose intensity is not affected by their distance from the radiation source.

With an x‐ray tube as radiation source the situation is completely different. In order to increase the low dose rate, the distance between source and experiment or potentially a patient has to be made as small as possible. However, this will lead to substantial photon absorption and scattering within the collimator and the photon flux will decrease according to the inverse square law with increasing distance from the radiation source. Moreover, the beam width and beam spacing will increase and geometric penumbra regions appear when moving away from the collimator. A parallel multislit collimator as employed for the microbeam experiments at the ESRF (Ref. 22) cannot be used.

We have addressed these challenges by designing the following technical setup. First, an x‐ray tube (HPX‐160‐11, Varian medical systems, Salt Lake City, USA) was mounted in an x‐ray cabinet (RS160 Cabinet, x‐strahl, Camberley, UK) such that the radiation beam is directed toward the ceiling of the cabinet. This dual focal spot x‐ray tube can be operated with a focal spot size of 0.4 mm at a maximum power of 800 W. The setup with an upward directed beam allows to place cells within millimeters from the collimator. There the beam divergence is expected to have a negligible effect on the beam shape and the dose rate will be highest.

**Figure 1:**
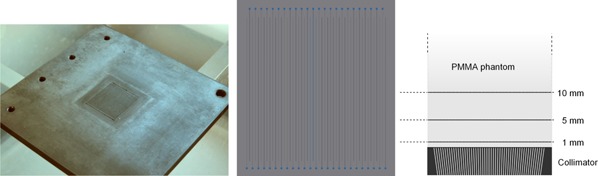
The multislit collimator used to shape microbeams with an x‐ray tube: photograph, front view and schematic profile. The latter also shows the PMMA phantom used for dosimetry on top of the collimator. Films were inserted 1, 5, and 10 mm above the upper collimator surface. The total size of the PMMA phantom was 100 × 100 × 50 mm^3^. For the *in vitro* exposures cells were placed 5 mm above the collimator surface inside the PMMA phantom.

The production of microbeams is accomplished by a carefully designed multislit collimator (Fig. 1) mounted at a distance of 70 mm from the radiation source, which is the minimal reasonably achievable distance in the cabinet. The collimator consists of 49 slits cut into a 5 mm thick tungsten plate, which absorbs 99.9% of the primary radiation. The 50 *μ*m wide and 20 mm long slits, separated by a distance of 400 *μ*m, are not perpendicular to the plate surface but are tilted to account for the beam divergence. Therefore, the slit spacings at the beam‐facing surface of the collimator of the plate are by 26.6 *μ*m smaller than at the top, which leads to an angle difference of around 18.3 arc min between adjacent beams. Only the beam aperture at the center of the radiation field does not have to account for this beam divergence.

The production of the collimator was carried out by state of the art milling and wire cutting (T&G engineering, Byfleet, UK). The tolerances were less than 1.5 arc min and 2.5 *μ*m for slit angles and distances, respectively. Slits are extended by 1.5 mm in an alternating manner where they start with a 0.3 mm diameter hole. These holes were necessary to get the wire in place and thread it through the plate before cutting. The collimator and its design are shown in Fig. 1.

**Figure 2:**
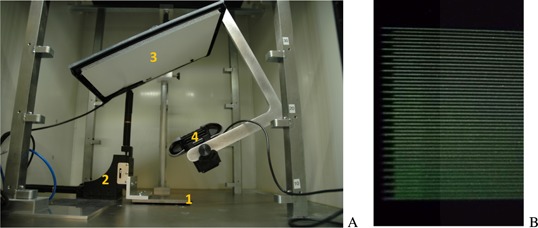
(A) shows a photograph of the experimental set‐up with multislit collimator (1), horizontal and vertical stepping motors (2), fluorescence screen (3), and webcam (4). A detector screenshot is shown in (B).

Finally, two motorized translational stages (Thorlabs Ltd., Ely, UK) are used to position the collimator with micrometer accuracy relative to the x‐ray tube. A horizontal drive stepping motor moves the collimator perpendicular to the central microbeam plane and a vertical drive stepping motor adjusts the distance between source and collimator surface. A fluorescence screen and a webcam were setup as a simple detector system to qualitatively monitor the dose rate behind the collimator. The setup and a typical detector image are shown in Fig. 2.

### 2.B. Dosimetry

Dosimetry was performed in a PMMA phantom of 100 × 100 × 50 mm^3^ size with Gafchromic® EBT3 films (Ashland, Covington, USA). EBT3 films are sensitive in a dose range between 0.1 and 40 Gy. Peak and valley dose measurements were separated. Exposure times were chosen such that peak or valley doses respectively fell into a range between 1 and 10 Gy. Readout of the exposed films was carried out with microscopy using the methods described previously.^23^ Calibration of the films was performed in an open field, i.e., without collimator, and reference dosimetry by a semiflex TW31010 and farmer TW30002‐1 ionization chamber (PTW, Darmstadt, Germany). Anode voltage, current, and filtering were kept constant between calibration and measurement. Dosimetry followed the TRS398 protocol,^24^ including detector calibration, temperature and pressure corrections, and the application of beam quality factors *k_Q_*.

For all experiments presented here the tube was driven at 160 kV and 5 mA using the small focal spot. Beams were filtered intrinsically by 0.8 mm beryllium and additionally by 1 mm aluminum. The measured halfvalue layer thickness of 0.31 mm copper was used to determine *k_Q_*.

### 2.C. *In vitro* experiments

As a proof‐of principle study, we performed an *in vitro* irradiation of pancreatic cancer cells at our microbeam facility and compared the results with an analogous experiment at the European Synchrotron. The aim was to show qualitative agreement and demonstrate the feasibility of microbeam experiments with a conventional x‐ray tube.

Pancreatic cancer cells, Panc1 (ATCC, Teddington, UK) were cultured in DMEM 1 g/l or 3.7 g/l glucose medium (ThermoFisher Scientific, Waltham, USA) with 10% FBS, 1% Pen/Strep Amphotericin B (Lonza biologics PLC, Slough, UK), 1% MEM NEAA, nonessential amino acids (PAN Biotech GmbH, Aidenbach, Germany), and 1% Gluta MAX (ThermoFisher Scientific). Cells were kept incubated at 100% humidity, 5% CO_2_, and 37 °C. Cells were either seeded on poly‐L‐lysine (Sigma Aldrich, St Louis, USA) coated coverslips and cultivated in Nunc Rectangular Dishes (ThermoFisher Scientific) or on T25 cell culture flasks.

For the synchrotron exposures cells were grown in T25 cell culture flasks. During the exposure the flasks were mounted in upright position behind a 5 mm thick PMMA layer. Film dosimetry showed that the PVDR is 22.0 ± 0.2. The exposure was chosen such that peak doses are at 80 Gy. Technical details of MRT exposures at the ESRF can, for example, be found in Bräuer‐Krisch *et al.* ^25^

In the exposures with the x‐ray cabinet Nunc Rectangular Dishes were placed in the solid water phantom used for dosimetry such that the total depth in the phantom and the distance from the upward (i.e., beam‐ward) pointing collimator surface are both 5 mm. Cells were exposed for 345 s to achieve a peak dose of 80 Gy. The PVDR was (21 ± 2) as shown in the following chapter. The MRT field size measured in all experiments 20 × 20 mm^2^ with a microbeam width and spacing of 50 and 400 *μ*m.

In both experiments the medium was changed before, but not after irradiation. Cells were fixated for 15 min with 4% formaldehyde solution for 1 and 12 h or 1 and 6 h after radiation exposure. Immunocytochemical staining was performed against the phosphorylated histone γH2AX. For that cells were treated 10 min with 0.3% Triton‐X (Sigma Aldrich) in PBS solution, kept for 60 min in blocking solution, 3% BSA (Sigma Aldrich), 0.3% Trion‐X in PBS and incubated overnight with a 1:1000 diluted antibody solution (Phospho‐Histone H2A.X, Alexa Fluor® 488 conjugate from Cell Signaling, Cambridge, UK). The signal was intensified by incubating with a 1:200 diluted secondary antibody solution for 60 min (AntiRabbit IgG, Alexa Fluor® 488 conjugate from Cell Signaling). Counterstaining of the cell nuclei was done with Hoechst 33342.

Epifluorescence microscopy was performed on an automated scanning microscope. For the x‐ray cabinet samples this was done on a Zeiss scanning microscope and for the synchrotron samples on a Nikon Zi‐HCS system. Coverslips were mounted on microscope slides before imaging. Image analysis and cell segmentation were done by the open source software CellProfiler^26^ and metafer (MetaSystems, Altlussheim, Germany).

## 3. RESULTS

### 3.A. Dosimetry

**Figure 3:**
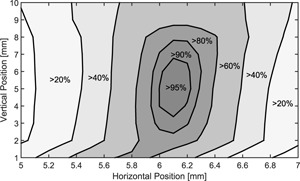
Relative output measured by the fluorescence screen detector depending on the horizontal and vertical motor position. The highest output is reached at 5 mm vertical and 6.1 mm horizontal position.

The fluorescence screen detector was employed to qualitatively monitor the dose rate behind the collimator. The dose rate depends on the horizontal and vertical collimator position. In Fig. 3 the mean intensity of all 49 beams is displayed as a function of the collimator location. In order to achieve more than 95% of the maximum dose rate, the horizontal position needs to be adjusted with ±100 *μ*m and the vertical position with ±1.5 mm accuracy. The highest output is achieved within 1 mm accuracy of the 70 mm focal spot to collimator distance used for the design of the collimator. In all further experiments the collimator was positioned at the point with maximum dose rate.

The results of film dosimetry are summarized in Table I. Peak dose is defined as the mean dose in the central 10 *μ*m of the peak and valley dose as the mean dose in the central 140 *μ*m of the trough. At 1 mm depth in solid water the average dose rate in the peak was measured to be (300 ± 20) mGy/s and a mean valley dose rate of (10.2 ± 0.5) mGy/s which yields a ratio between peak and valley dose (PVDR) of (30 ± 3). The PVDR rapidly decreases with depth. Measurements at 10 mm depth gave a PVDR of just 15.5 ± 1.5. The rapid degradation of the PVDR with depth is caused by a growing geometric penumbra of the divergent x‐ray beam.

**Table I:** Peak and valley doses measured by film dosimetry at 1, 5, and 10 mm depth in a solid water phantom (Fig. 1). PVDRs in this domain are similar to PVDRs expected at the ESRF for a 20 × 20 mm^2^ microbeam field.

Depth (mm)	1	5	10
Peak (mGy/s)	300 ± 20	230 ± 10	170 ± 10
Valley (mGy/s)	10.2 ± 0.5	11.2 ± 0.5	10.7 ± 0.5
PVDR	30 ± 3	21 ± 2	15.5 ± 1.5

**Figure 4:**
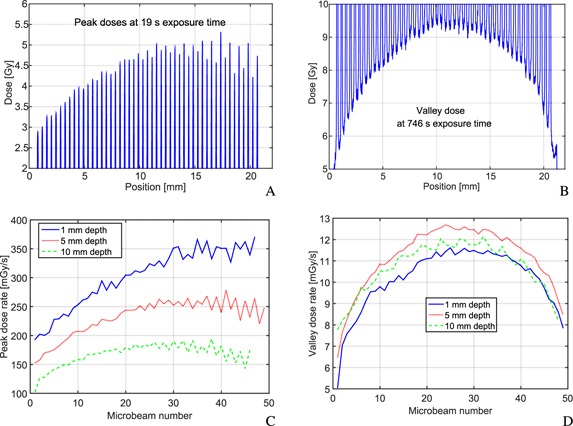
Results of film dosimetry and microscope readout are displayed for the produced 20 × 20 mm^2^ microbeam fields. The top row presents profiles at 5 mm depth. Due to the limited dose range of the EBT3 films, (A) was obtained with 19 s exposure time and (B) with 746 s exposure time. The bottom row compares peak (C) and valley (D) dose rates at 1, 5, and 10 mm depth (Fig. 1).

Measured microbeam profiles at 5 mm depth and peak and valley dose profiles at 1, 5, and 10 mm depth are presented in Fig. 4. Valley dose rates at all three depths are very similar and profiles across the field resemble those measured at the ESRF. For the x‐ray tube the valley dose at the high beam number edge (right edge of the radiation field in Fig. 4) of the field has dropped to around 68% of the maximum valley dose. Similar variations are observed for these field sizes for synchrotron produced microbeams. However, the valley dose rate profile is not symmetric around the field center. For the peak doses an increase from small to large peak numbers is observable. Especially at 1 mm depth the peak dose increases by more than 40% across the field. In greater depths beam intensities become more balanced.

At high beam numbers (right edge of the profiles) beam intensities vary from microbeam to microbeam in an alternating manner. At 5 mm depth beams with odd numbers are slightly higher in their intensity than beams with even numbers. This pattern is also observable on the fluorescence screen and is probably related to inaccuracies in the manufacturing process of the collimator since the wire cutting direction did also change in an alternating manner. A small offset in the slit inclination could therefore cause such an intensity pattern.

**Figure 5:**
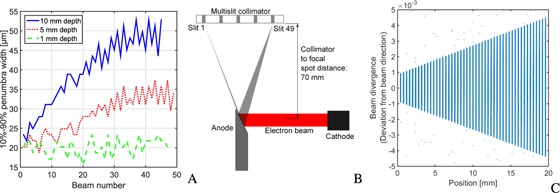
(A) shows the width of the microbeam penumbras depending on the beam number at 1, 5, and 10 mm depth. The explanation for the penumbra variation across the field at 5 and 10 mm depth can be understood from the tube‐collimator alignment shown in (B). The focal spot appears under different angles. The effect on the beam divergence is demonstrated in (C), where the deviation from the beam axis (assuming a point source) is plotted over the photon position directly behind the collimator. Each dot in the graph corresponds to a photon trajectory in a Monte Carlo simulation of the source‐collimator setup.

The PVDR resembles closely the values obtained at the synchrotron. To further compare the quality of the produced microbeams, film dosimetry results were used to measure the beam penumbras. The beam penumbra width is arbitrarily defined as the spatial range where the dose falls off from 90% to 10% of the peak dose. In Fig. 5(A), the beam penumbra width is visualized for the three examined depths across the microbeam field. At 1 mm depth the beam penumbra is constantly around 20 *μ*m wide across the field. At 5 and 10 mm depth the beam penumbra width is around 20 *μ*m for beam 1. With increasing beam number the penumbras become wider and reach approximately 33 and 48 *μ*m at the other end of the field at 5 and 10 mm depth, respectively.

The alignment of the collimator with the x‐ray tube causes the variation in the beam penumbra across the microbeam field as illustrated in Fig. 5(B). Due to the tilted anode the apparent focal spot size seen at the collimator depends on the slit position. The focal spot appears under a wider angle for larger beam numbers. This directly affects the beam divergence. In contrast to a point source, where all photons passing through a collimator slit *i* have the same direction *α_i_*, an extended source will produce photons that pass the slit in an angle interval around *α_i_*, *α_i_* ± Δ*α_i_*. Figure 5(C) shows results of a Monte Carlo simulation of the x‐ray tube source and the multislit collimator with the above specifications. The beam divergence Δ*α* is plotted against the photon position directly behind the multislit collimator. Each point in the graph corresponds to an individually simulated photon.

### 3.B. *In vitro* experiments

**Figure 6:**
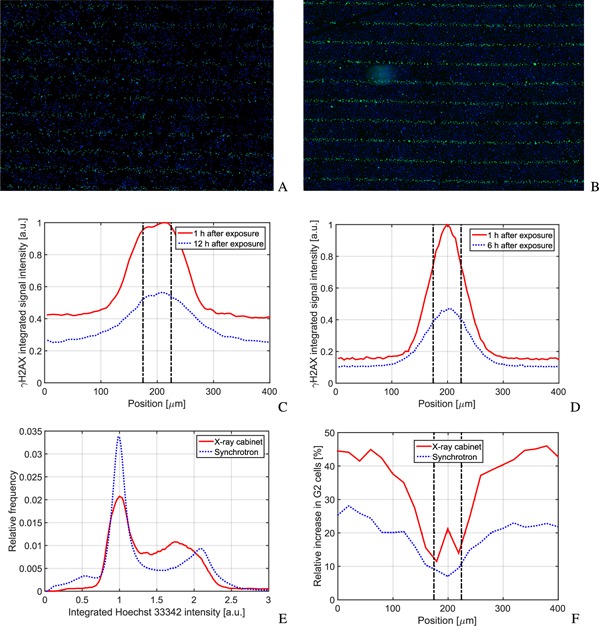
(A) and (B) show a detail of a fluorescence image taken 1 h after radiation exposure with the x‐ray cabinet and the ESRF, respectively. The green GFP‐γH2AX (emission at 509 nm) and blue Hoechst 33342 (emission at 461 nm) channel are combined for the images. The image measure in both cases is (*W* × *H*) 6.49 × 4.55 mm^2^ in size. (C) and (D) show a comparison of the γH2AX signal intensity across a single microbeam, 1 and 12 h after radiation exposure at the x‐ray cabinet and 1 and 6 h at the synchrotron. The Hoechst 33342 signal can be used to analyze the distribution of cells in the cell cycle. A histogram 1 h after exposure is shown in (E). Cells clearly cluster in the G1 and G2 phase. The distribution is independent of the position in the field. This is changing, however, at later time points. (F) shows the relative increase in G2 cells across an individual microbeam. Cells in the valley accumulate stronger in the G2 phase than cells in the peak. In (C), (D), and (F) the position of the microbeam is indicated by vertical dashed lines.

Examples of acquired fluorescence microscopy images for the experiment at the x‐ray cabinet and the European Synchrotron are shown in Figs. 6(A) and 6(B). Cells in the microbeam path can visually clearly be distinguished from cells in the valley. The signal intensity of the green labeled phosphorylated γH2AX histone is much higher. The cell density in A is around 200 mm^−2^ and in B around 600 mm^−2^.

A quantitative evaluation of the γH2AX signal intensity per cell is shown in Figs. 6(C) and 6(D), at two time points after radiation exposure across one microbeam. With time, the intensity of the γH2AX signal decreases in both valley and peak. In the x‐ray cabinet experiment, the signal decreases by 45% and 40% in the peak and in the valley, respectively. In the beam penumbra region, the signal decrease is lower at around 30%. Therefore the width of the penumbra, defined again as range where the signal lies between 10% and 90% of the peak, becomes wider. One hour after radiation exposure it is 50 *μ*m and 12 h after exposure 90 *μ*m. These are both significantly larger than the dose beam penumbra.

For the synchrotron exposure, the signal reduction is even more pronounced with 60% in the peak. The reduction in the valley is just 28% and in the beam penumbra regions 22%. The γH2AX signal in the synchrotron experiment follows the microbeam profile more closely and the beam peak appears sharper. However, due to differences in the experimental condition, such as cell densities, imaging devices, time points, and plating surfaces, detailed quantitative comparisons cannot be validated in this first proof‐of‐principle experiment.

An interesting behavior of the cells in the microbeam field can be seen in the bottom row of Fig. 6. Hoechst 33342 fluorescently stains DNA and the signal intensity depends on the amount of DNA in the cell nucleus. Consequently, cells in G1 phase before DNA replication have a lower signal intensity than cells in G2 phase after DNA replication. One hour after radiation exposure the distribution of cells in the cell cycle has not significantly changed. Across the microbeam field the relative cell count histogram over the integrated Hoechst 33342 intensity yields independently of the position the distribution shown in Fig. 6(E). Clearly the two peaks of G1 and G2 phase cells can be distinguished. Figure 6(F) shows the relative increase of cells in the G2 phase across a microbeam. Cells are accumulating in the G2 phase, due to the G2 cell cycle arrest. Interestingly this process is much stronger at lower doses in the valley than at higher doses in the peak.

The observation was made for both experiments, at the European Synchrotron and with the x‐ray cabinet. Again quantitative conclusions are difficult to draw. The definition of G1 and G2 phase cells is arbitrary and based on different imaging systems. Moreover, the cell cycle is also affected by the cell density, which was different in both experiments.

## 4. DISCUSSION

It was demonstrated that microbeams for *in vitro* experiments can be produced by conventional x‐ray tubes at reasonable dose rates. With the presented setup, dose rates of up to 300 mGy/s were reached at a beam width of 50 *μ*m and a beam‐to‐beam spacing of 400 *μ*m. Beam‐to‐beam spacing and beam width correspond to the specifications envisaged for future clinical trials at the European Synchrotron (ESRF) in Grenoble.^27^ Dose rates for MRT at the European Synchrotron reach between 8 and 16 kGy/s^25^ and are orders of magnitude higher than at an x‐ray tube. However, the synchrotron beam at the ESRF has a maximum height of just 520 *μ*m. Therefore the target has to be scanned through the beam to produce higher fields, while the x‐ray tube produced microbeam field can be applied statically. For nonmoving targets, such as *in vitro* cells, the absorbed radiation dose should not be affected by the lower dose rate, although dose rate effects might start to play a role.^28^

The dose distribution in a 20 × 20 mm^2^ microbeam field produced in the x‐ray cabinet is very similar to the dose distribution obtained in preclinical experiments at the European Synchrotron. The peak to valley dose ratio ranged between 15.5 and 30 in measurements at the x‐ray cabinet, similar to values measured and simulated at the ESRF.^29^, ^30^ Close to the collimator beam, penumbras are sharp and approximately equal to those measured at the ESRF. The 10% to 90% penumbra definition yields 29 *μ*m penumbra width in film dosimetry measurements at the ESRF.^23^ However, x‐ray tube generated microbeams had clearly wider penumbras when moving away from the collimator. At 10 mm distance they attained 50 *μ*m.

While x‐ray tube generated microbeams are dosimetrically comparable to microbeams produced at the European Synchrotrons in terms of beam penumbras and PVDRs, the achieved uniformity across the microbeam field is lower. Peak doses at the ESRF vary by just around 5% (Ref. 29) in a 20 × 20 mm^2^ field, whereas peak variations around the mean for the x‐ray tube produced microbeams are as high as 25% in 5 mm depth. There are two main reasons for the fluctuations. The dominating effect is the alignment between tube and collimator [Fig. 5(B)]. Each microbeam is produced with a different projected focal spot size and has a different beam spectrum due to the heel effect. If beams are labeled as in Fig. 5(B), then the projected focal spot size is small at small beam numbers, leading to narrower beam penumbras. However, the heel effect strongly affects beams with small beam numbers. These beams therefore have a lower photon fluence and a harder energy spectrum. While this leads to lower peak doses close to the collimator, the lower beam divergence at low beam numbers and absorption of low energy photons at large beam numbers counterbalance the effect at greater distances and depths. The second reason for peak dose variations is manufacturing inaccuracies that lead to the alternating high and low peak dose pattern (Fig. 4). The uniformity of the beam penumbras could be considerably increased if the collimator was rotated by 90° around the central beam axis. The setup will be changed accordingly in future versions. However, variations in the peak dose are expected to have a negligible effect on tissue and cell survival, since the fraction of surviving cells in the peak region is extremely low at high peak doses.

In a pilot experiment, it was demonstrated that the developed setup is suitable to perform *in vitro* experiments. For similar experimental settings, pancreatic cancer cells (Panc1) were irradiated with a 20 × 20 mm^2^ microbeam field with 80 Gy peak dose, 50 *μ*m beam width, 400 *μ*m beam spacing, and a PVDR of around 22 at the European Synchrotron and with the developed setup. The phosphorylation of the histone H2AX was measured and the G2‐cell cycle arrest examined after microbeam irradiation. Qualitatively experimental results are similar in both experiments. Especially the G2 cell cycle arrest in the valley region is remarkable. Since cells of the Panc1 cell line have a mutated p53 gene, a G1 cell cycle arrest of cells in the microbeam peak can be ruled out. However, it should be noted that these results have to be further validated and more experiments will be carried out with the developed source to investigate the *in vitro* behavior of cells after microbeam treatment.

## 5. CONCLUSION

Research in microbeam radiation therapy is so far almost exclusively restricted to large synchrotrons. In this work, it was shown that conventional x‐ray tubes could provide an alternative radiation source. With a specially designed multislit collimator, microbeams were produced for *in vitro* experiments at a dose rate of up to 300 mGy/s. In a first proof‐of‐principle study, the feasibility of the developed source for the *in vitro* experiments is demonstrated and first results show that for both radiation sources the G2 cell cycle arrest is less pronounced in the peak than in the valley. The authors envisage a modification of their method and the development of a small animal microbeam irradiator in the near future. For a clinical application the limited dose rate is still the main obstacle to be overcome.

## ACKNOWLEDGMENT

This work was supported by Cancer Research UK [C57410/A21787].

## CONFLICT OF INTEREST DISCLOSURE

The authors have no COI to report.
